# 1-Ethyl-3-methyl­quinoxalin-2(1*H*)-one

**DOI:** 10.1107/S1600536808033989

**Published:** 2008-10-31

**Authors:** Hanane Benzeid, Laure Vendier, Youssef Ramli, Bernard Garrigues, El Mokhtar Essassi

**Affiliations:** a Université Mohamed V, Département de Chimie, Laboratoire de Chimie Organique Hétérocyclique, Pôle de Compétences Pharmacochimie, BP 1014, Avenue Ibn Batouta, Rabat, Morocco; b Laboratoire de Chimie de Coordination, 205 Route de Narbonne, 3, 1077, Toulouse Cedex 04, France; c Université Paul Sabatier, Hétérochimie Fondamentale et Appliquée, UMR 5069, 118 Route de Narbonne, 31062 Toulouse Cedex, France

## Abstract

The asymmetric unit of the title compound, C_11_H_12_N_2_O, contains two independent mol­ecules. In the crystal structure, inter­molecular C—H⋯O hydrogen bonds link the mol­ecules. There are π–π contacts between the quinoxaline rings [centroid–centroid distances = 3.446 (2), 3.665 (2), 3.645 (3) and 3.815 (3) Å]. There also exist C—H⋯π contacts between the methyl groups and the quinoxaline rings.

## Related literature

For general background, see: Amin (2003 ▶); Boutti & Lecolier (1975 ▶); Milos & John (1981 ▶); Rose *et al.* (1990 ▶); Salman *et al.* (2007 ▶); Kotharkar & Shinde (2006 ▶); Vishnu *et al.* (2006 ▶). For related literature, see: Nikolaenko & Munro (2004 ▶). For bond-length data, see: Allen *et al.* (1987 ▶).
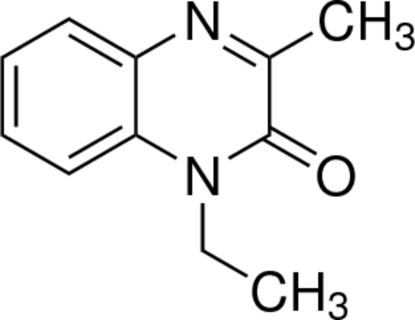


## Experimental

### 

#### Crystal data

C_11_H_12_N_2_O
*M* *_r_* = 188.23
Triclinic, 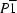

*a* = 7.4101 (6) Å
*b* = 9.1405 (8) Å
*c* = 14.2960 (12) Å
α = 84.976 (7)°
β = 78.717 (7)°
γ = 88.137 (7)°
*V* = 945.82 (14) Å^3^
*Z* = 4
Mo *K*α radiation
μ = 0.09 mm^−1^
*T* = 180 K
0.18 × 0.13 × 0.07 mm

#### Data collection

Oxford Diffraction Xcalibur diffractometer
Absorption correction: multi-scan (*CrysAlis RED*; Oxford Diffraction, 2007 ▶) *T*_min_ = 0.988, *T*_max_ = 0.991
7441 measured reflections
3865 independent reflections
2874 reflections with *I* > 2σ(*I*)
*R*_int_ = 0.021

#### Refinement

*R*[*F* ^2^ > 2σ(*F* ^2^)] = 0.039
*wR*(*F* ^2^) = 0.111
*S* = 1.06
3865 reflections
257 parameters
H-atom parameters constrained
Δρ_max_ = 0.17 e Å^−3^
Δρ_min_ = −0.25 e Å^−3^

### 

Data collection: *CrysAlis CCD* (Oxford Diffraction, 2007 ▶); cell refinement: *CrysAlis RED* (Oxford Diffraction, 2007 ▶); data reduction: *CrysAlis RED*; program(s) used to solve structure: *SIR92* (Altomare *et al.*, 1994 ▶); program(s) used to refine structure: *SHELXL97* (Sheldrick, 2008 ▶); molecular graphics: *ORTEP-3 for Windows* (Farrugia, 1997 ▶) and *PLATON* (Spek, 2003 ▶); software used to prepare material for publication: *WinGX* (Farrugia, 1999 ▶) and *PLATON*.

## Supplementary Material

Crystal structure: contains datablocks global, I. DOI: 10.1107/S1600536808033989/hk2546sup1.cif

Structure factors: contains datablocks I. DOI: 10.1107/S1600536808033989/hk2546Isup2.hkl

Additional supplementary materials:  crystallographic information; 3D view; checkCIF report

## Figures and Tables

**Table 1:** Hydrogen-bond geometry (Å, °)

*D*—H⋯*A*	*D*—H	H⋯*A*	*D*⋯*A*	*D*—H⋯*A*
C7—H7⋯O2^i^	0.93	2.43	3.291 (3)	154
C17—H17⋯O1	0.93	2.46	3.301 (3)	151
C22—H22*C*⋯*Cg*4^ii^	0.96	2.71	3.516 (3)	142

Symmetry codes: (i) 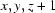; (ii) 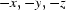. *Cg*4 is the centroid of the C14–C19 ring.

**Fig. 1.**
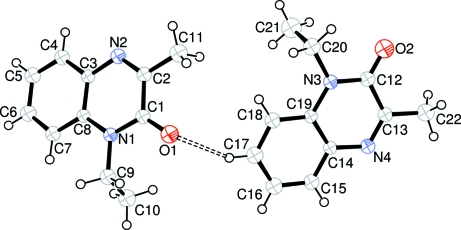
The molecular structure of the title molecule, with the atom-numbering scheme. Hydrogen bond is shown as dashed line.

**Fig. 2.**
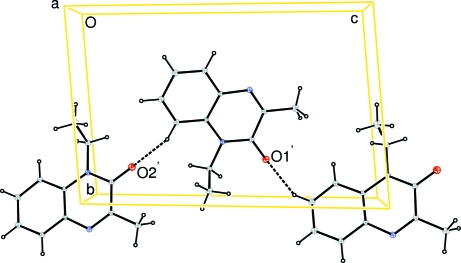
A partial packing diagram. Hydrogen bonds are shown as dashed lines [symmetry code: (') -x, -y, -z].

## supplementary crystallographic information

### Comment

The quinoxaline derivatives have great importance in scientific research for their biological properties. It is well known that quinoxaline have antibacterial (Kotharkar & Shinde, 2006; Salman *et al.*, 2007) and antifungal (Vishnu *et al.*, 2006) activities. They are also used for colorimetry metal detection (Amin, 2003) and as oil stabilizant (Boutti & Lecolier, 1975). Likewise several patents describe them as hair azo dyes (Rose *et al.*, 1990) and pigments (Milos & John, 1981). We report herein, the synthesis and crystal structure of the title compound.

The asymmetric unit of the title compound (Fig. 1), contains two independent molecules. The bond lengths (Allen *et al.*,  1987) and angles are within normal ranges. The intramolecular C—H···O hydrogen bond (Table 1) links the molecules.

In the crystal structure, intra- and intermolecular C-H···O hydrogen bonds (Table 1) link the molecules (Fig. 2), in which they may be effective in the stabilization of the structure. The π—π contacts between the quinoxaline rings, Cg1···Cg1^i^, Cg1···Cg3^ii^, Cg2···Cg2^iii^ and Cg2···Cg4^iv^ [symmetry codes: (i) 1 - x, 1 - y, 1 - z; (ii) -x, 1 - y, 1 - z; (iii) -x, -y, -z; (iv) 1 - x, -y, -z, where Cg1, Cg2, Cg3 and Cg4 are the centroids of the rings A (N1/N2/C1-C3/C8), B (N3/N4/C12-C14/C19), C (C3-C8) and D (C14-C19), respectively] may further stabilize the structure, with centroid-centroid distances of 3.446 (2), 3.665 (2), 3.645 (3) and 3.815 (3) Å, respectively. There also exist C—H···π contacts (Table 1) between the methyl groups and rings C and D.

### Experimental

To a solution of 3-methylquinoxalin-2(1*H*)-one (Nikolaenko & Munro, 2004) (1 g, 6.22 mmol) in dimethylformamide (20 ml), was added ethylbromide (0.67 ml, 6.22 mmol), K_2_CO_3_ (1 g, 7.46 mmol) and a catalytic quantity of tetrabutylammoniumbromide. The mixture was stirred at room temperature for 24 h. The solution was filtered to remove the salts. The solvent was removed under reduced pressure. The residue was crystallized in ethanol to afford the title compound as yellow crystals.

### Refinement

H atoms were positioned geometrically, with C-H = 0.93, 0.97 and 0.96 Å for aromatic, methylene and methyl H, respectively, and constrained to ride on their parent atoms with U_iso_(H) = xU_eq_(C), where x = 1.5 for methyl H and x = 1.2 for all other H atoms.

### Crystal data

**Table:**

C_11_H_12_N_2_O	*Z* = 4
*M**_r_* = 188.23	*F*(000) = 400
Triclinic, *P*1	*D*_x_ = 1.322 Mg m^−^^3^
Hall symbol: -P 1	Mo *K*α radiation, λ = 0.71073 Å
*a* = 7.4101 (6) Å	Cell parameters from 4794 reflections
*b* = 9.1405 (8) Å	θ = 2.8–31.9°
*c* = 14.2960 (12) Å	µ = 0.09 mm^−^^1^
α = 84.976 (7)°	*T* = 180 K
β = 78.717 (7)°	Block, colorless
γ = 88.137 (7)°	0.18 × 0.13 × 0.07 mm
*V* = 945.82 (14) Å^3^	

### Data collection

**Table:**

Oxford Diffraction Xcalibur diffractometer	3865 independent reflections
Radiation source: fine-focus sealed tube	2874 reflections with *I* > 2σ(*I*)
graphite	*R*_int_ = 0.021
φ and ω scans	θ_max_ = 26.4°, θ_min_ = 2.8°
Absorption correction: multi-scan (CrysAlis RED; Oxford Diffraction, 2007)	*h* = −9→8
*T*_min_ = 0.988, *T*_max_ = 0.991	*k* = −11→11
7441 measured reflections	*l* = −14→17

### Refinement

**Table:**

Refinement on *F*^2^	Primary atom site location: structure-invariant direct methods
Least-squares matrix: full	Secondary atom site location: difference Fourier map
*R*[*F*^2^ > 2σ(*F*^2^)] = 0.039	Hydrogen site location: inferred from neighbouring sites
*wR*(*F*^2^) = 0.111	H-atom parameters constrained
*S* = 1.06	*w* = 1/[σ^2^(*F*_o_^2^) + (0.0636*P*)^2^ + 0.0861*P*] where *P* = (*F*_o_^2^ + 2*F*_c_^2^)/3
3865 reflections	(Δ/σ)_max_ < 0.001
257 parameters	Δρ_max_ = 0.17 e Å^−^^3^
0 restraints	Δρ_min_ = −0.25 e Å^−^^3^

### Special details

**Table:**

Geometry. All e.s.d.'s (except the e.s.d. in the dihedral angle between two l.s. planes) are estimated using the full covariance matrix. The cell e.s.d.'s are taken into account individually in the estimation of e.s.d.'s in distances, angles and torsion angles; correlations between e.s.d.'s in cell parameters are only used when they are defined by crystal symmetry. An approximate (isotropic) treatment of cell e.s.d.'s is used for estimating e.s.d.'s involving l.s. planes.
Refinement. Refinement of *F*^2^ against ALL reflections. The weighted *R*-factor *wR* and goodness of fit *S* are based on *F*^2^, conventional *R*-factors *R* are based on *F*, with *F* set to zero for negative *F*^2^. The threshold expression of *F*^2^ > σ(*F*^2^) is used only for calculating *R*-factors(gt) *etc*. and is not relevant to the choice of reflections for refinement. *R*-factors based on *F*^2^ are statistically about twice as large as those based on *F*, and *R*- factors based on ALL data will be even larger.

### Fractional atomic coordinates and isotropic or equivalent isotropic displacement parameters (Å^2^)

**Table:**

	*x*	*y*	*z*	*U*_iso_*/*U*_eq_	
O1	0.46438 (13)	0.21858 (11)	0.37756 (7)	0.0375 (3)	
O2	0.28053 (14)	0.17912 (12)	−0.17004 (7)	0.0438 (3)	
N1	0.33532 (14)	0.31106 (11)	0.51763 (7)	0.0244 (2)	
N2	0.28293 (14)	0.57622 (11)	0.41274 (8)	0.0279 (3)	
N3	0.30886 (13)	0.14773 (11)	−0.01436 (7)	0.0258 (2)	
N4	0.19176 (14)	−0.14053 (12)	−0.01083 (8)	0.0274 (3)	
C1	0.39293 (17)	0.32233 (14)	0.42044 (9)	0.0267 (3)	
C2	0.36552 (17)	0.46723 (14)	0.37053 (9)	0.0274 (3)	
C3	0.21391 (16)	0.55680 (13)	0.51001 (9)	0.0237 (3)	
C4	0.11835 (18)	0.67263 (14)	0.55460 (10)	0.0300 (3)	
H4	0.1021	0.7601	0.5186	0.036*	
C5	0.04770 (19)	0.66048 (15)	0.65059 (10)	0.0342 (3)	
H5	−0.0161	0.7388	0.6799	0.041*	
C6	0.07254 (19)	0.52952 (16)	0.70377 (10)	0.0344 (3)	
H6	0.0251	0.521	0.7692	0.041*	
C7	0.16544 (18)	0.41247 (15)	0.66197 (9)	0.0296 (3)	
H7	0.1795	0.3253	0.6987	0.036*	
C8	0.23840 (16)	0.42472 (13)	0.56451 (9)	0.0228 (3)	
C9	0.37008 (19)	0.16990 (14)	0.56915 (10)	0.0311 (3)	
H9A	0.3822	0.1864	0.6337	0.037*	
H9B	0.4852	0.1276	0.5373	0.037*	
C10	0.2174 (2)	0.06358 (15)	0.57337 (11)	0.0386 (3)	
H10A	0.1059	0.1005	0.6106	0.058*	
H10B	0.2494	−0.0298	0.6025	0.058*	
H10C	0.1995	0.0522	0.5097	0.058*	
C11	0.4365 (2)	0.48358 (17)	0.26554 (10)	0.0396 (3)	
H11A	0.3503	0.4428	0.2332	0.059*	
H11B	0.5527	0.4326	0.251	0.059*	
H11C	0.4525	0.5858	0.2446	0.059*	
C12	0.26924 (17)	0.09898 (15)	−0.09593 (9)	0.0287 (3)	
C13	0.21489 (17)	−0.05575 (15)	−0.08903 (9)	0.0279 (3)	
C14	0.22409 (16)	−0.08415 (13)	0.07134 (9)	0.0236 (3)	
C15	0.19919 (18)	−0.17617 (15)	0.15556 (9)	0.0306 (3)	
H15	0.157	−0.2709	0.1556	0.037*	
C16	0.23571 (19)	−0.12955 (17)	0.23841 (10)	0.0361 (3)	
H16	0.2194	−0.1921	0.2942	0.043*	
C17	0.29710 (19)	0.01175 (17)	0.23769 (10)	0.0358 (3)	
H17	0.3223	0.044	0.2936	0.043*	
C18	0.32169 (17)	0.10564 (15)	0.15571 (10)	0.0308 (3)	
H18	0.3624	0.2006	0.1567	0.037*	
C19	0.28562 (16)	0.05865 (13)	0.07122 (8)	0.0232 (3)	
C20	0.37012 (19)	0.29997 (14)	−0.01979 (11)	0.0359 (3)	
H20A	0.432	0.3291	−0.0847	0.043*	
H20B	0.4577	0.3061	0.022	0.043*	
C21	0.2105 (2)	0.40401 (17)	0.00908 (14)	0.0525 (4)	
H21A	0.1243	0.399	−0.0326	0.079*	
H21B	0.2549	0.5024	0.0044	0.079*	
H21C	0.1508	0.3768	0.0739	0.079*	
C22	0.1875 (2)	−0.11426 (18)	−0.17904 (10)	0.0399 (4)	
H22A	0.1497	−0.2146	−0.166	0.06*	
H22B	0.3009	−0.109	−0.2251	0.06*	
H22C	0.0943	−0.0569	−0.2042	0.06*	

### Atomic displacement parameters (Å^2^)

**Table:**

	*U*^11^	*U*^22^	*U*^33^	*U*^12^	*U*^13^	*U*^23^
O1	0.0409 (6)	0.0364 (5)	0.0342 (5)	0.0068 (4)	−0.0026 (4)	−0.0111 (4)
O2	0.0460 (6)	0.0514 (6)	0.0287 (5)	0.0052 (5)	−0.0026 (4)	0.0131 (5)
N1	0.0263 (5)	0.0214 (5)	0.0260 (6)	0.0021 (4)	−0.0066 (4)	−0.0019 (4)
N2	0.0302 (6)	0.0253 (6)	0.0291 (6)	−0.0059 (4)	−0.0083 (5)	0.0009 (4)
N3	0.0222 (5)	0.0247 (5)	0.0273 (6)	0.0021 (4)	0.0011 (4)	0.0009 (4)
N4	0.0246 (6)	0.0304 (6)	0.0266 (6)	0.0030 (4)	−0.0023 (4)	−0.0061 (5)
C1	0.0221 (6)	0.0302 (7)	0.0285 (7)	−0.0015 (5)	−0.0051 (5)	−0.0058 (5)
C2	0.0257 (6)	0.0310 (7)	0.0263 (7)	−0.0063 (5)	−0.0065 (5)	−0.0013 (5)
C3	0.0219 (6)	0.0229 (6)	0.0283 (6)	−0.0039 (5)	−0.0086 (5)	−0.0024 (5)
C4	0.0321 (7)	0.0212 (6)	0.0396 (8)	−0.0005 (5)	−0.0136 (6)	−0.0036 (5)
C5	0.0326 (7)	0.0302 (7)	0.0419 (8)	0.0034 (6)	−0.0077 (6)	−0.0154 (6)
C6	0.0359 (8)	0.0396 (8)	0.0277 (7)	−0.0012 (6)	−0.0033 (6)	−0.0094 (6)
C7	0.0331 (7)	0.0295 (7)	0.0265 (7)	0.0001 (6)	−0.0071 (5)	−0.0006 (5)
C8	0.0212 (6)	0.0223 (6)	0.0268 (6)	−0.0013 (5)	−0.0075 (5)	−0.0046 (5)
C9	0.0369 (8)	0.0251 (7)	0.0312 (7)	0.0088 (6)	−0.0081 (6)	−0.0015 (5)
C10	0.0475 (9)	0.0257 (7)	0.0394 (8)	0.0006 (6)	−0.0026 (7)	0.0013 (6)
C11	0.0436 (8)	0.0447 (8)	0.0284 (7)	−0.0039 (7)	−0.0023 (6)	0.0003 (6)
C12	0.0216 (6)	0.0377 (7)	0.0234 (7)	0.0069 (5)	0.0010 (5)	0.0014 (6)
C13	0.0202 (6)	0.0381 (7)	0.0242 (7)	0.0066 (5)	−0.0014 (5)	−0.0060 (5)
C14	0.0193 (6)	0.0268 (6)	0.0235 (6)	0.0041 (5)	−0.0010 (5)	−0.0041 (5)
C15	0.0295 (7)	0.0287 (7)	0.0301 (7)	0.0041 (5)	−0.0002 (5)	0.0028 (5)
C16	0.0351 (8)	0.0462 (9)	0.0235 (7)	0.0105 (6)	−0.0019 (6)	0.0039 (6)
C17	0.0319 (7)	0.0518 (9)	0.0251 (7)	0.0110 (6)	−0.0078 (5)	−0.0102 (6)
C18	0.0263 (7)	0.0332 (7)	0.0341 (7)	0.0034 (5)	−0.0058 (5)	−0.0117 (6)
C19	0.0171 (6)	0.0265 (6)	0.0238 (6)	0.0054 (5)	0.0001 (5)	−0.0017 (5)
C20	0.0325 (7)	0.0263 (7)	0.0445 (8)	−0.0010 (6)	0.0015 (6)	0.0022 (6)
C21	0.0460 (9)	0.0285 (8)	0.0800 (13)	0.0068 (7)	−0.0047 (8)	−0.0070 (8)
C22	0.0340 (8)	0.0588 (10)	0.0284 (7)	0.0060 (7)	−0.0064 (6)	−0.0136 (7)

### Geometric parameters (Å, °)

**Table:**

C1—O1	1.2238 (15)	C12—O2	1.2245 (15)
C1—N1	1.3671 (16)	C12—N3	1.3705 (17)
C1—C2	1.4759 (18)	C12—C13	1.473 (2)
C2—N2	1.2854 (16)	C13—N4	1.2891 (17)
C2—C11	1.4862 (18)	C13—C22	1.4876 (19)
C3—N2	1.3825 (17)	C14—N4	1.3882 (16)
C3—C4	1.3862 (17)	C14—C15	1.3918 (18)
C3—C8	1.4049 (17)	C14—C19	1.3963 (18)
C4—C5	1.366 (2)	C15—C16	1.371 (2)
C4—H4	0.93	C15—H15	0.93
C5—C6	1.388 (2)	C16—C17	1.382 (2)
C5—H5	0.93	C16—H16	0.93
C6—C7	1.3707 (19)	C17—C18	1.3761 (19)
C6—H6	0.93	C17—H17	0.93
C7—C8	1.3885 (18)	C18—C19	1.3926 (18)
C7—H7	0.93	C18—H18	0.93
C8—N1	1.3891 (15)	C19—N3	1.3934 (16)
C9—N1	1.4696 (15)	C20—N3	1.4687 (17)
C9—H9A	0.97	C20—H20A	0.97
C9—H9B	0.97	C20—H20B	0.97
C10—C9	1.5041 (19)	C21—C20	1.509 (2)
C10—H10A	0.96	C21—H21A	0.96
C10—H10B	0.96	C21—H21B	0.96
C10—H10C	0.96	C21—H21C	0.96
C11—H11A	0.96	C22—H22A	0.96
C11—H11B	0.96	C22—H22B	0.96
C11—H11C	0.96	C22—H22C	0.96
			
O1—C1—N1	121.88 (12)	N4—C13—C22	119.61 (13)
O1—C1—C2	122.05 (12)	C12—C13—C22	116.56 (12)
N1—C1—C2	116.07 (11)	N4—C14—C15	118.06 (12)
N2—C2—C1	123.59 (11)	N4—C14—C19	122.33 (11)
N2—C2—C11	119.68 (12)	C15—C14—C19	119.57 (12)
C1—C2—C11	116.73 (12)	C16—C15—C14	121.14 (13)
N2—C3—C4	118.34 (11)	C16—C15—H15	119.4
N2—C3—C8	122.24 (11)	C14—C15—H15	119.4
C4—C3—C8	119.42 (12)	C15—C16—C17	118.97 (13)
C5—C4—C3	121.10 (12)	C15—C16—H16	120.5
C5—C4—H4	119.5	C17—C16—H16	120.5
C3—C4—H4	119.5	C18—C17—C16	121.23 (13)
C4—C5—C6	119.04 (12)	C18—C17—H17	119.4
C4—C5—H5	120.5	C16—C17—H17	119.4
C6—C5—H5	120.5	C17—C18—C19	120.05 (13)
C7—C6—C5	121.44 (13)	C17—C18—H18	120
C7—C6—H6	119.3	C19—C18—H18	120
C5—C6—H6	119.3	C18—C19—N3	122.96 (12)
C6—C7—C8	119.68 (12)	C18—C19—C14	119.04 (12)
C6—C7—H7	120.2	N3—C19—C14	118.00 (11)
C8—C7—H7	120.2	N3—C20—C21	111.52 (11)
C7—C8—N1	122.76 (11)	N3—C20—H20A	109.3
C7—C8—C3	119.32 (11)	C21—C20—H20A	109.3
N1—C8—C3	117.92 (11)	N3—C20—H20B	109.3
N1—C9—C10	111.68 (11)	C21—C20—H20B	109.3
N1—C9—H9A	109.3	H20A—C20—H20B	108
C10—C9—H9A	109.3	C20—C21—H21A	109.5
N1—C9—H9B	109.3	C20—C21—H21B	109.5
C10—C9—H9B	109.3	H21A—C21—H21B	109.5
H9A—C9—H9B	107.9	C20—C21—H21C	109.5
C9—C10—H10A	109.5	H21A—C21—H21C	109.5
C9—C10—H10B	109.5	H21B—C21—H21C	109.5
H10A—C10—H10B	109.5	C13—C22—H22A	109.5
C9—C10—H10C	109.5	C13—C22—H22B	109.5
H10A—C10—H10C	109.5	H22A—C22—H22B	109.5
H10B—C10—H10C	109.5	C13—C22—H22C	109.5
C2—C11—H11A	109.5	H22A—C22—H22C	109.5
C2—C11—H11B	109.5	H22B—C22—H22C	109.5
H11A—C11—H11B	109.5	C1—N1—C8	121.35 (10)
C2—C11—H11C	109.5	C1—N1—C9	116.87 (10)
H11A—C11—H11C	109.5	C8—N1—C9	121.66 (10)
H11B—C11—H11C	109.5	C2—N2—C3	118.47 (11)
O2—C12—N3	121.81 (13)	C12—N3—C19	121.53 (11)
O2—C12—C13	122.35 (13)	C12—N3—C20	117.23 (11)
N3—C12—C13	115.83 (11)	C19—N3—C20	121.20 (11)
N4—C13—C12	123.82 (12)	C13—N4—C14	118.27 (11)

### Hydrogen-bond geometry (Å, °)

**Table:**

*D*—H···*A*	*D*—H	H···*A*	*D*···*A*	*D*—H···*A*
C7—H7···O2^i^	0.93	2.43	3.291 (3)	154
C17—H17···O1	0.93	2.46	3.301 (3)	151
C11—H11B···Cg3^ii^	0.96	3.34	3.893 (3)	119
C22—H22C···Cg4^iii^	0.96	2.71	3.516 (3)	142

Symmetry codes: (i) *x*, *y*, *z*+1; (ii) −*x*+1, −*y*+1, −*z*+1; (iii) −*x*, −*y*, −*z*.

## References

[bb1] Allen, F. H., Kennard, O., Watson, D. G., Brammer, L., Orpen, A. G. & Taylor, R. (1987). *J. Chem. Soc. Perkin Trans. 2*, pp. S1–19.

[bb2] Altomare, A., Cascarano, G., Giacovazzo, C., Guagliardi, A., Burla, M. C., Polidori, G. & Camalli, M. (1994). *J. Appl. Cryst.* **27**, 435.

[bb3] Amin, A. S. (2003). *Spectrochim. Acta Part A*, **59**, 1025–1033. 10.1016/s1386-1425(02)00259-7 12633719

[bb4] Boutti, D. & Lecolier, S. (1975). Fr. Patent 2 249 879.

[bb5] Farrugia, L. J. (1997). *J. Appl. Cryst.* **30**, 565.

[bb6] Farrugia, L. J. (1999). *J. Appl. Cryst.* **32**, 837–838.

[bb12] Kotharkar, S. A. & Shinde, D. B. (2006). *Bioorg. Med. Chem. Lett.* **16**, 6181–6184. 10.1016/j.bmcl.2006.09.040 17027265

[bb7] Milos, B. & John, F. C. (1981). *Dyes Pigm.* **2**, 215–217.

[bb8] Nikolaenko, I. V. & Munro, O. Q. (2004). *Acta Cryst.* E**60**, o92–o94.

[bb9] Oxford Diffraction (2007). *CrysAlis CCD* and *CrysAlis RED* Oxford Diffraction Ltd, Abingdon, Oxfordshire, England.

[bb10] Rose, D., Lieske, E., Hoeffkes, H. & Henkel, K.-Ga. (1990). German Offen. DE 3 212 825.

[bb11] Salman, A. K., Kishwar, S. & Zaheer, K. (2007). *Eur. J. Med. Chem.* **42**, 103–108.

[bb13] Sheldrick, G. M. (2008). *Acta Cryst.* A**64**, 112–122. 10.1107/S0108767307043930 18156677

[bb14] Spek, A. L. (2003). *J. Appl. Cryst.* **36**, 7–13.

[bb15] Vishnu, K. T., Dharmendra, B. Y., Hardesh, K. M., Ashok, K. C. & Praveen, K. S. (2006). *Bioorg. Med. Chem. Lett.* **14**, 6120–6126.

